# Analysis of the temperature- and fluence-dependent magnetic stress in laser-excited SrRuO_3_

**DOI:** 10.1063/4.0000072

**Published:** 2021-03-24

**Authors:** M. Mattern, J.-E. Pudell, G. Laskin, A. von Reppert, M. Bargheer

**Affiliations:** 1 Institut für Physik und Astronomie, Universität Potsdam, Karl-Liebknecht-Str. 24-25, 14476 Potsdam, Germany; 2 Helmholtz Zentrum Berlin, Albert-Einstein-Str. 15, 12489 Berlin, Germany; 3 Max Planck Institute for Solid State Research, Heisenbergstrasse 1, 70569 Stuttgart, Germany

## Abstract

We use ultrafast x-ray diffraction to investigate the effect of expansive phononic and contractive magnetic stress driving the picosecond strain response of a metallic perovskite SrRuO_3_ thin film upon femtosecond laser excitation. We exemplify how the anisotropic bulk equilibrium thermal expansion can be used to predict the response of the thin film to ultrafast deposition of energy. It is key to consider that the laterally homogeneous laser excitation changes the strain response compared to the near-equilibrium thermal expansion because the balanced in-plane stresses suppress the Poisson stress on the picosecond timescale. We find a very large negative Grüneisen constant describing the large contractive stress imposed by a small amount of energy in the spin system. The temperature and fluence dependence of the strain response for a double-pulse excitation scheme demonstrates the saturation of the magnetic stress in the high-fluence regime.

## I. INTRODUCTION

Magnetic stresses in laser-excited materials are interesting because they potentially allow one to manipulate the ultrafast, nanoscopic strain response by changing macroscopic parameters such as temperature, laser fluence, or applied external fields. The identification of the magnetic contributions, however, requires a dissection of the total stress into the contributions from different energy reservoirs that also include phonons and electronic excitations. Additional stresses may arise from an unbalanced in-plane expansion that couples, via the Poisson effect, to the out-of-plane direction.

In general, the laser-induced strain represents the deterministic elastic response to the stress that originates from the energy transfer to different degrees of freedom in a solid. In the case of metals without magnetic order, this stress originates from the energy transfer to the electrons and phonons.^1,2^ In typical pump-probe experiments, the laser pulse is initially absorbed by the valence electrons, and the subsequent transfer of energy to phonons determines the depth- and time-dependent stress. A useful concept to describe the stress is based on subsystem-specific Grüneisen constants that linearly relate the deposited energy density in the energy reservoirs to the induced stress.^3,4^ In contrast to the prediction of a simple two-temperature model, mode-specific electron-phonon coupling can result in non-thermal phonon distributions for tens of picoseconds described by mode-specific temperatures.^5–7^ The non-thermal occupation of phonon modes may influence the induced stress under the condition of different mode-specific Grüneisen constants.^8–10^ However, in many materials, the induced stress can be approximated by a macroscopic Grüneisen constant because also typical non-equilibrium distributions that deviate from the Bose- or Fermi-distributions average over the various degrees of freedom that receive the energy. In recent publications, subsystem-specific Grüneisen constants have been used to rationalize the strain response to electron-phonon stresses^11–14^ and ultrafast energy transport in metallic heterostructures.^15,16^ Historically, the Grüneisen constant describes the simultaneous contribution of quantum excitations in various phonon modes to both the heat capacity and the thermal expansion.^17^ This thermodynamic concept was further generalized to electronic^18^ and magnetic stresses,^4,18–20^ negative thermal expansion,^21^ and anisotropic expansion.^4,22,23^

Magnetic excitations in solids represent an additional energy reservoir and are expected to exert a stress upon energy transfer. The resulting magnetostriction changes the atomic arrangement both in equilibrium^24–28^ and in ultrafast experiments.^8,29–31^ In SrRuO_3_ (SRO), temperature-dependent experiments show a direction-dependent invar behavior due to an anisotropic contractive magnetic stress.^32–34^ One of the first time-resolved studies of ultrafast magnetostriction demonstrated that the strain of SRO layers within a SrRuO_3_-SrTiO_3_-superlattice is reduced by a contribution that acts already within the first picosecond and follows the squared magnetization $M^{2}(T)$.^29^ Recent studies in heavy rare earth metals introduce magnetic Grüneisen constants to discuss the spatiotemporal stress that arises from magnetic excitations and its contribution to laser-induced strain dynamics.^35–37^ An analysis of the stress contributions to the strain response of the magnetic perovskite SRO using the concept of Grüneisen constants was so far not attempted although this material is frequently used as opto-acoustic transducer in femtosecond laser-excitation experiments.^38–41^

Here, we present a discussion of the stress contributions to the temperature-dependent ultrafast strain response that is measured by ultrafast x-ray diffraction (UXRD) on a 19 nm thin SRO film for two different fluence regimes. We analyze the expansion driven by two delayed pump pulses as a function of the temperature-dependent magnetic order. For both fluences, we observe a significant decrease in the expansion below the Curie temperature $T_{C}$ because the contractive magnetic stress counteracts the expansive phonon stress. For the lower fluence, the total stress exhibits a step-like decrease if the initial temperature is lowered below the phase transition. This decrease is continuous for the higher fluence and mimics the $M^{2}(T)$ dependence. We explain both observations by a Grüneisen model, which relates the ultrafast expansion to the subsystem-specific heat capacities and Grüneisen constants that are anisotropic for the spin system and isotropic for the combined electron-phonon system. These Grüneisen constants are extracted from the available thermal expansion of bulk SRO by subtracting the Poisson contribution that arises from the in-plane expansion.

The observed fluence dependence is then rationalized by the saturation of the magnetic stress, which is especially pronounced in the strain response to a second, approximately 10 ps delayed, pump pulse. In the limit of small fluences, the temperature dependence of the stress is given by the temperature-dependent magnetic heat capacity, whereas in the case of a transient heating above $T_{C}$ (high fluence limit), it is determined by the integral of the remaining magnetic heat capacity.

We emphasize that our modeled stress allows extracting the relative energy dissipation by the spin system and the electron-phonon system. Here, we do not attempt to gain insights into microscopic non-equilibrium processes that may influence details of the strain dynamics in the first picosecond. This has advantages and disadvantages: While the model is universally applicable for predicting a first-order approximation of ultrafast lattice dynamics from static equilibrium data in complex magnetic systems, it does not yield details of the magnetization dynamics.

This paper consists of three main parts: In Sec. II, we present the relevant equilibrium thermodynamic properties from which we extract the anisotropic magnetic and phononic Grüneisen constants of SRO along the out-of-plane direction of the thin film. Section III presents the experimental results on the picosecond strain response of a thin SRO film. Section IV provides the modeling of the strain response based on the anisotropic Grüneisen constants from Sec. II and discusses the extracted fluence- and temperature-dependent magnetic stress contribution.

## II. EQUILIBRIUM PROPERTIES

### A. Sample characterization

**FIG. 1.**
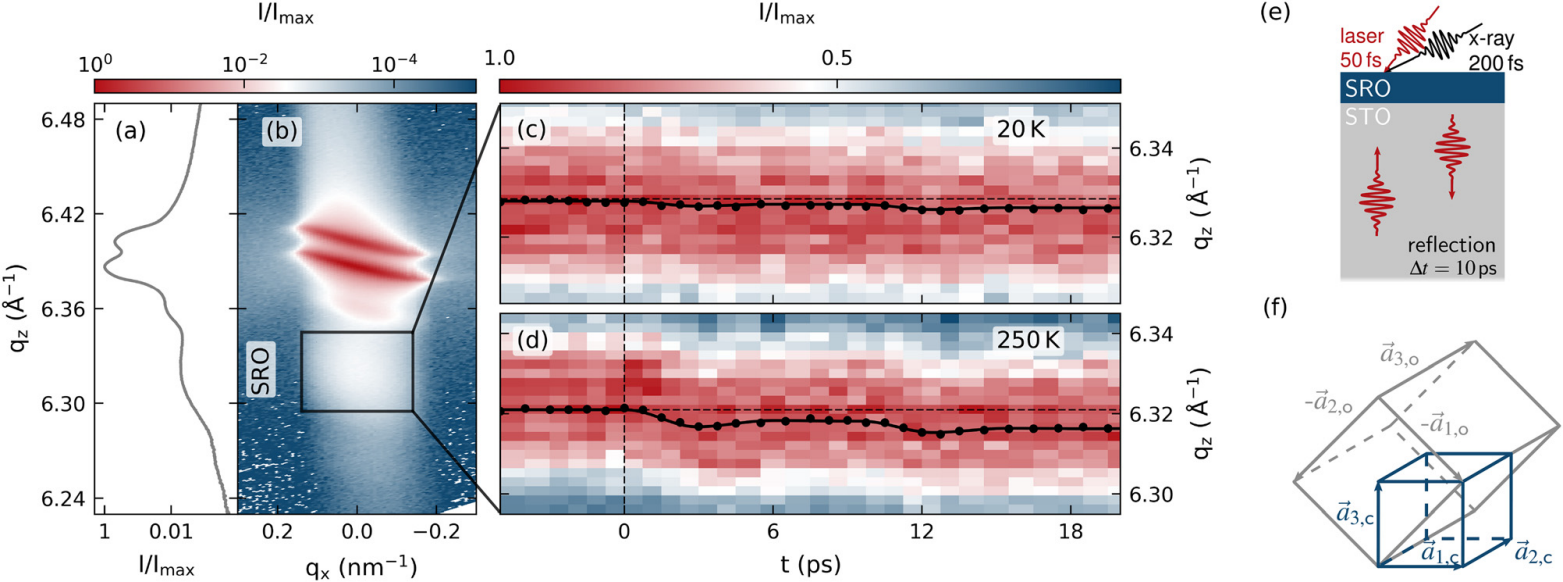
Characterization of the sample and the measurement procedure for the time-resolved strain evaluation: (a) projection of the reciprocal space onto the *q_z_*-direction obtained by the integration of the reciprocal space map shown in (b) along the *q_x_*-direction. The ${\left(004\right)}_{c}$ SRO-Bragg peak is found at $q_{z}=6.32\;{Å}^{-1}$ as a shoulder of the STO substrate peak. (c) Transient shift of the SRO peak upon laser excitation at low fluence $F=1.4\;{\text{mJcm}}^{-2}$ and low temperature T=20 K. Its center-of-mass is indicated by black symbols, (d) same for T=250 K. (e) The second delayed excitation at 10 ps arises from the reflection of the partially transmitted pump pulse from the backside of the substrate. (f) Sketches of the orientation of the orthorhombic ${\left[110\right]}_{o}$-oriented SRO unit cell in relation to the pseudocubic unit cell that is aligned along the out-of-plane direction of the SRO film.

The investigated sample shown in Fig. 1(e) consists of a continuous 19 nm thin SRO film grown on a SrTiO_3_ (STO) substrate using pulsed laser deposition.^42,43^ The sub-percent in-plane lattice mismatch between the pseudocubic unit cells of the film and the substrate leads to a coherent growth of ${\left(001\right)}_{c}$-oriented SRO on ${\left(001\right)}_{c}$-oriented STO^44^ as it is typical for functional perovskite heterostructures.^45^ The orientations of the SRO unit cell in orthorhombic and pseudocubic notation in the transducer-substrate system are shown in Fig. 1(f). In agreement with the literature,^46,47^ the orthorhombic ${\left[110\right]}_{o}$ unit cell direction of SRO points out-of-plane. The reciprocal space map (RSM) measured by a microfocus Cu-$K_{\alpha }$ x-ray source shown in Fig. 1(b) displays the barely separated ${\left(004\right)}_{c}$ Bragg peaks of the SRO film and the substrate as diffraction maxima. Note, the complicated shape of the substrate Bragg peak corresponds to the instrument function of two rotated two-dimensional Gaussian functions.^48^ Integrating the RSM along the *q_x_*-direction shows the SRO peak as a shoulder of the substrate peak. The small contribution at $6.36\;{Å}^{-1}$ hints at a strained surface layer of the STO substrate.^49^ In the time-resolved experiment discussed later, we exploit that the Bragg peak position of the SRO layer shifts upon laser excitation as shown in Figs. 1(c) and 1(d). The transient shift of the center-of-mass (COM) along the *q_z_* direction (black symbols) yields the average, time-dependent strain $\left(\eta_{3}(t)=\frac{\Delta d(t)}{d_{0}}\right)$ in the SRO film from the relation $q_{z}=\frac{2\pi n}{d}$ between the out-of-plane lattice spacing *d* and the reciprocal coordinate *q_z_* with the diffraction order *n*. The thin film only partially absorbs the 800 nm laser pulse, so that the reflection from the backside of the 0.6 mm thick, transparent STO substrate leads to a delayed second excitation as shown in Fig. 1(e).

In the following, we provide an overview of the temperature-dependent thermodynamic and magnetic properties of SRO. We determine the phononic and magnetic anisotropic Grüneisen constant from equilibrium thermal expansion and the subsystem-separated heat capacity, which are used in the analysis of the time-resolved measurements presented in Sec. III.

### B. Temperature-dependent properties of SRO

The metallic perovskite SRO changes from the paramagnetic state to ferromagnetic order below its Curie temperature $T_{C}=160\;K$.^50,51^ For thin films, $T_{C}$ is reduced to 150 K by the tetragonal distortion of the pseudocubic unit cell via a substrate-induced stress, which is especially pronounced for SRO-nanodots.^43,44^ The strong link between the lattice and magnetic degrees of freedom in SRO is supported by the observation of an unusually high magnetocrystalline anisotropy, which indicates a strong spin–orbit interaction.^52,53^ In the case of a thin film on STO, the magnetization of SRO points in the ${\left[010\right]}_{o}$-direction at the transition temperature, i.e., it is at $45^{{}^{\circ}}$ with respect to the surface normal of the pseudocubic film. This angle changes to 30° with decreasing temperature.^54,55^ Furthermore, the magnetic order below $T_{C}$ induces a freezing of the oxygen octahedral rotation as observed in neutron diffraction experiments.^33,34^

**FIG. 2.**
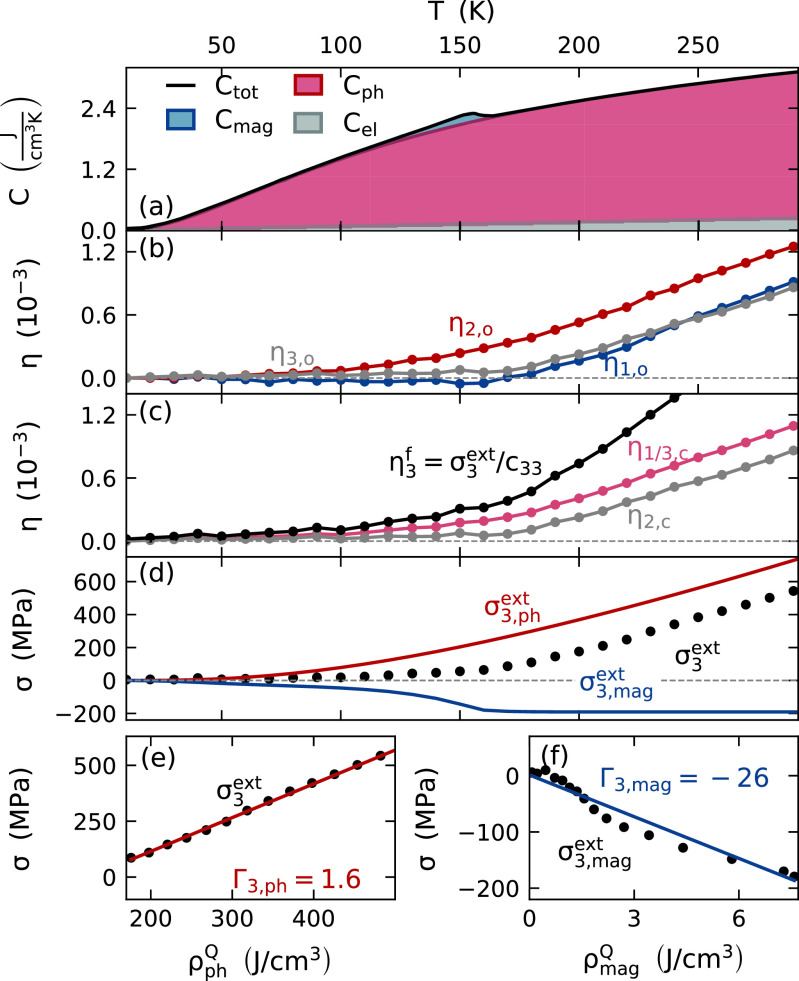
Determination of the out-of-plane Grüneisen constants using temperature-dependent thermodynamic properties of SRO: (a) subsystem contributions to the total bulk heat capacity^56^ provide the temperature-dependent energy distribution $\rho_{r}^{Q}(T)$ by integration. The orthorhombic lattice strains^32–34^ (b) define the pseudocubic strains (c) according to the orientation in the thin film. Here, $\eta_{3}^{f}=\sigma_{3}^{\text{ext}}/c_{33}$ denotes the response under the condition of fixed in-plane lattice constants to (d) the external stress $\sigma_{3}^{\text{ext}}(T)$. Panel (e) shows the total stress $\sigma_{3}^{\text{ext}}$ as the function of the phonon energy density $\rho_{ph}^{Q}(T)$ above 170 K, where the magnetic heat capacity has no contribution (panel a). The linear slope yields the phonon Grüneisen constant $\Gamma_{3,\text{ph}}=1.6$ which determines the phonon stress $\sigma_{3,\text{ph}}^{\text{ext}}(T)$ (red line in panel d) for the entire temperature range. Panel (f) displays the resulting magnetic stress contribution $\sigma_{3,\text{mag}}^{\text{ext}}(T)=\sigma_{3}^{\text{ext}}(T)-\sigma_{3,\text{ph}}^{\text{ext}}(T)$ as function of the magnetic energy density. The slope of the linear fit yields the giant magnetic Grüneisen constant $\Gamma_{3,\text{mag}}=-26$.

This pronounced interplay of the structure and the itinerant ferromagnetism of the hybridized Ru-4d and O-2p electrons^50,57^ reduces the expansion of bulk SRO in the ferromagnetic phase.^32–34^ The temperature-dependent orthorhombic lattice strains $\left(\eta (T)=\frac{\Delta d(T)}{d(20\;\;K)}\right)$ along the ${\left[100\right]}_{o}$ and ${\left[001\right]}_{o}$ directions depicted in Fig. 2(b) are essentially constant up to $T_{C}$, whereas the strain along ${\left[010\right]}_{o}$ shows a positive thermal expansion. The vanishing strain along ${\left[100\right]}_{o}$ and ${\left[001\right]}_{o}$ indicates an anisotropic compensation of the thermal expansion by a contractive magnetic stress that vanishes above *T_C_*. The corresponding pseudocubic equilibrium lattice strains in Fig. 2(c) are defined by the orientation of the orthorhombic unit cell depicted in Fig. 1(f). By the definition of the pseudocubic unit cell, the strains $\eta_{1,c}=\eta_{3,c}$ are equal, and $\eta_{2,c}$ is different.

The magnetic origin of the expansion anomaly is corroborated by the heat capacity,^56^ which also yields a clear macroscopic signature of the magnetic phase transition in form of a peak at the transition temperature. We separate the total bulk heat capacity at constant volume $C_{\text{tot}}$ into the contribution of electrons, phonons, and the magnetic subsystem. The Sommerfeld model is used to approximate the electronic heat capacity to $C_{\text{el}}=\gamma_{S}T$ with the Sommerfeld constant $\gamma_{S}=30\;\;\text{mJ}\;\;{\text{mol}}^{-1}\;K^{-2}=0.8\;\;\text{mJ}\;\;{\text{cm}}^{-3}\;K^{-2}$.^56^ Instead of using a Debye model, the phonon contribution $C_{\text{ph}}$ is calculated as the weighted sum of the heat capacities of the oxides SrO^58^ and RuO,^59^ which is fitted to the total heat capacity above $T_{C}$ reduced by the electron contribution. This is necessary because the widely dispersed phonon density of states makes the Debye temperature $T_{D}$ of SRO strongly temperature-dependent.^56,60^ The magnetic contribution results from the difference between the total heat capacity and the sum of electronic and phononic contributions. The separated subsystem contributions for bulk SRO shown in Fig. 2(a) are used to determine the Grüneisen constants in Sec. II C.

As mentioned earlier, in thin films, the substrate-induced deformation of the unit cell reduces the Curie temperature to $T_{C}=150\;\;K$, and the decrease in this deformation near the surface broadens the phase transition. We account for these changes by determining the magnetic heat capacity of the thin film from the magnetization measurements via the molecular field approximation.^61^ We relate the magnetization of a comparable film^43,54^ to the magnetic heat capacity $C_{\text{mag}}^{\text{film}}\propto M\frac{dM}{dT}$. The amplitude is determined by fitting the temperature-dependent shape resulting from Bloch's $T^{\frac{3}{2}}$ law to the bulk magnetic heat capacity below 50 K. The resulting heat capacity determines the magnetic excitations in the analysis of the pump-probe experiment in Sec. IV.

### C. Anisotropic subsystem Grüneisen constants

The thermal expansion $\eta_{i}(T)$ of the pseudocubic unit cell of SRO is the elastic response to a stress $\sigma_{i}=\sigma_{i}^{\text{ext}}-\sigma_{i}^{\text{poi}}$ originating from the excitation of phonons and spins and additional Poisson stresses $\sigma_{i}^{\text{poi}}$ induced by the thermal expansion along the orthogonal directions $\eta_{j\neq i}$. The direction-dependent thermal expansion of SRO indicates underlying anisotropic stresses $\sigma_{i}^{\text{ext}}$. To capture the anisotropic stress driving the anisotropic thermal expansion, we introduce anisotropic Grüneisen constants of the subsystems *r*, which linearly relate the deposited energy density $\rho_{r}^{Q}$ as a scalar quantity to a direction-dependent uni-axial stress $\sigma_{i,r}^{\text{ext}}$.^4,23^

In the following, we limit the discussion to the out-of-plane stress, which drives the out-of-plane strain response that we observe in the pump-probe experiment by UXRD. The total out-of-plane stress is the superposition of the stresses $\sigma_{3}^{\text{ext}}=\sum_{r}\sigma_{3,r}^{\text{ext}}$, which are linearly related to the energy density stored in each subsystem $\rho_{r}^{Q}$ by a subsystem-specific Grüneisen constant $\Gamma_{3,r}$. In a cubic crystal, this Grüneisen constant is given by^4,23^

$$\Gamma_{3,r}=\frac{c_{33}}{C_{r}(T)}(\alpha_{3,r}+\frac{c_{13}}{c_{33}}(\alpha_{1,r}+\alpha_{2,r})),$$ (1)

where *c*_13_ and *c*_33_ are the elastic tensor elements in Voigt notation and *α_i_* is the cubic thermal expansion coefficients in thermal equilibrium. For a sufficiently small temperature change ΔT, Eq. (1) transforms to

$$\begin{array}{c} \Gamma_{3,r}=\frac{c_{33}}{\rho_{r}^{Q}}\left(\eta_{3,r}(T)+\frac{c_{13}}{c_{33}}\left(\eta_{1,r}(T)+\eta_{2,r}(T)\right)\right)\\ =\frac{c_{33}\eta_{3,r}^{f}(T)}{\rho_{r}^{Q}}=\frac{\sigma_{3,r}^{\text{ext}}}{\rho_{r}^{Q}}, \end{array}$$ (2)

using the thermal expansion coefficients $\alpha_{i}=\partial \eta_{i}/\partial T$ and the energy density $\rho_{r}^{Q}(T)=\int_{T}^{T+\Delta T}C_{r}(T\prime )dT\prime$. In consideration of the Poisson stress $\sigma_{i}^{\text{poi}}=\sum_{j\neq i}c_{ij}\eta_{j}$,^31^ the bracket in Eq. (2) describes the strain $\eta_{3,r}^{f}$ for fixed in-plane lattice dimensions. Thus, the Grüneisen constant $\Gamma_{3,r}$ linearly relates the energy density $\rho_{r}^{Q}$ to the external out-of-plane stress $\sigma_{3,r}^{\text{ext}}$.

Following Eq. (2), we calculate the thermal expansion $\eta_{3}^{f}$ driven by the uni-axial external stress $\sigma_{3}^{\text{ext}}$, which is depicted in Fig. 2(c) as black line. We use the elastic constants $c_{13}=132\;\text{GPa}$ and $c_{33}=252\;\text{GPa}$ of bulk SRO.^62^ The corresponding temperature-dependent stress $\sigma_{3}^{\text{ext}}=c_{33}\eta_{3}^{f}$ is the superposition of stress contributions from phonons and magnetic excitations. The decomposition into these two stress contributions using subsystem-specific Grüneisen constants is shown in Figs. 2(e) and 2(f). Above 170 K, the total stress originates exclusively from phonons because the magnetic heat capacity converges to zero.

In Fig. 2(e), this phonon-related stress is depicted as a function of the energy density stored in the combined electron-phonon system $\rho_{\text{ph}}^{Q}=\int_{20\;K}^{T}C_{\text{el}}(T\prime )+C_{\text{ph}}(T\prime )dT\prime$. The nonlinear temperature dependence of the stress measured at discrete temperatures in Fig. 2(e) can be read from Fig. 2(d). The linear slope of the total stress on the energy density beyond 170 K yields the phononic Grüneisen constant $\Gamma_{3,\text{ph}}=1.6$ according to Eq. (2). This temperature-independent Grüneisen constant allows for determining the phononic stress over the full temperature range as depicted by the red solid line in Fig. 2(d). The difference between the phonon and the total stress defines the magnetic stress contribution, which is depicted in Fig. 2(f) as function of the magnetic energy density $\rho_{\text{mag}}^{Q}$ obtained from Fig. 2(a) by integration. Disregarding small deviations from a linear function that may result from uncertainties in the extraction of the small magnetic heat capacity, we again fit the linear slope to obtain the out-of-plane magnetic Grüneisen constant $\Gamma_{3,\text{mag}}=-26$. Its very large negative value indicates that even small amounts of energy in the magnetic excitations of SRO lead to a pronounced out-of-plane contraction, which partially compensates the normal thermal expansion. The finite integral of the magnetic heat capacity limits the energy storable in the magnetic degrees of freedom that results in a saturation of the magnetic stress above 170 K.

The resulting separation of the total stress into the contribution of phonons and magnetic excitations based on the Grüneisen constants is depicted in Fig. 2(d) and provides insight into the origin of the temperature-dependent expansion. Although the magnetic excitations and phonons lead to non-linear functions of their thermal expansion $\alpha_{r}(T)$ and their heat capacity $C_{r}(T)$ (Fig. 2), the situation can be approximated by two simple numbers, the Grüneisen constants, which linearly map energy density in the energy reservoirs to stress and—according to Hooke's law—also to strain.

## III. TIME-RESOLVED EXPERIMENTS

In the UXRD experiment, the sample is excited by a p-polarized, 100 fs-long optical laser pulse with a central wavelength of 800 nm at a repetition rate of 1 kHz. The incident fluence *F* is calculated from the laser power and the two-dimensional Gaussian beam footprint of 0.9 mm×1.1 mm full-width half-maximum (FWHM) assuming a top-hat profile and in consideration of the incident angle of 19° with respect to the surface normal. The temperature-independent optical penetration depth of 52 nm (Ref. 63) exceeds the layer thickness. Thus, the SRO layer is nearly homogeneously excited, and a fraction of the pump pulse is transmitted through it. The backreflection from the sample holder leads to a second excitation after 10 ps with a fluence of 0.6·F [see Fig. 1(e)].

Using UXRD, we observe the laser-induced, out-of-plane strain $\eta_{3}(t)$, which is driven by an out-of-plane stress gradient $\partial \sigma_{3}^{\text{ext}}/\partial x_{3}$ according to the inhomogeneous elastic wave equation.^1,2^ The in-plane strain vanishes, due to the laterally homogeneous excitation of the probed volume by a pump pulse with a beam footprint that is large in comparison to the probe-spot. Since the in-plane expansion is prohibited on picosecond timescales, the Poisson stress $\sigma_{3}^{\text{poi}}$ vanishes and the strain response is exclusively driven by the uni-axial stress $\sigma_{3}^{\text{ext}}$ described by the previously derived Grüneisen constants $\Gamma_{3,r}$. The absence of the Poisson stress on the picosecond timescale qualitatively changes the strain response with respect to equilibrium thermal expansion as indicated by the difference between the black and the red line in Fig. 2(c).

**FIG. 3.**
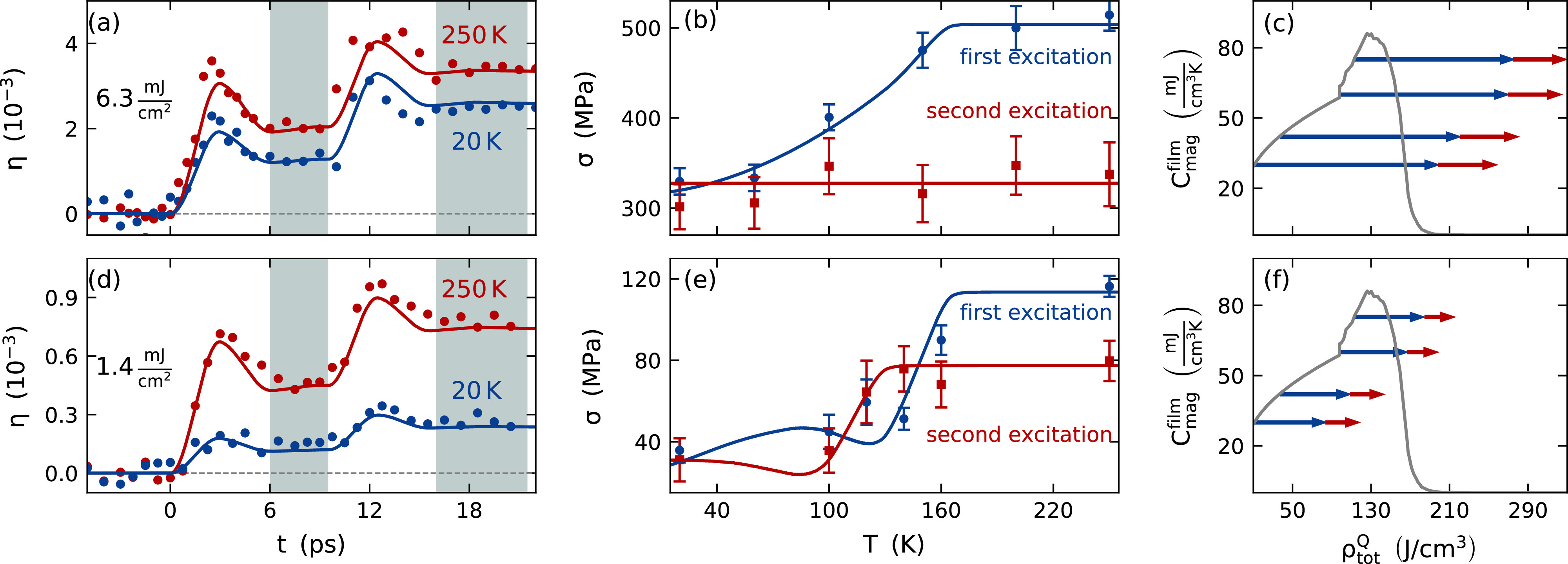
Temperature-dependent strain response of SRO in the high (a)–(c) and low fluence regime (d)–(f): the transient strain (symbols) measured by UXRD for $F=6.3\;\;\text{mJ}\;\;{\text{cm}}^{-2}$ (a) and $F=1.4\;\;\text{mJ}\;\;{\text{cm}}^{-2}$ (d) exhibits a temperature-independent temporal shape as illustrated for two representative data sets for each fluence. The general strain response is well described by a one-dimensional linear-chain model of masses and springs (solid lines).^70^ Panels (b) and (e) summarize the average stress induced by the first and second excitation as blue and red data points, respectively. We extract the stress from the mean strain of the shaded regions from 6 to 9 ps and 16 to 20 ps. The standard deviation of the transient strain at these delays determines the uncertainty. The total lattice stress decreases for initial sample temperatures below $T_{C}$ for the first excitation (blue) in both fluence regimes, whereas the stress after the second (red) excitation remains nearly constant in the high fluence regime shown in (b). The underlying excitation of the magnetic subsystem is sketched in panels (c) and (f) that show the equilibrium magnetic heat capacity as function of the equilibrium energy density. The laser-induced increase in the energy density by the first (blue) and second (red) laser pulse is sketched by horizontal arrows for different initial temperatures. If the energy density is large enough, the thin film is demagnetized and we observe a saturation of the magnetic stress. We note that the form of the specific heat of the spin system may change in nonequilibrium, and the figure should be interpreted qualitatively.

The time-resolved out-of-plane strain is probed by 200 fs long X-ray pulses with a photon energy of approximately 8 keV that are generated by a laser-based table-top plasma X-ray source (PXS).^64^ We measure for each pump-probe delay a reciprocal space map and determine the position of the SRO Bragg peak along *q_z_*. This requires the subtraction of the underlying substrate peak [Fig. 1(a)] that is considered as a static Lorentz profile describing the diffraction of the crystal truncation rod of the STO substrate. The center-of-mass analysis of the SRO Bragg peak yields the transient scattering at $q_{z}(t)$ [black symbols in Figs. 1(c) and 1(d)] that determines the transient strain by $\eta_{3}(t)=\frac{-\Delta q_{z}(t)}{q_{z}}$. The measured transient strain for the two excitation fluences of $F=6.3\;\;\text{mJ}\;\;{\text{cm}}^{-2}$ and $1.4\;\;\text{mJ}\;\;{\text{cm}}^{-2}$ for two representative initial temperatures T=250  K and T=20  K is summarized in Figs. 3(a) and 3(d).

For each pump pulse, we observe a fast expansion, which reaches its maximum 3 ps after the excitation and decreases within the following 3 ps to a certain expansion level. This observation is independent of the initial temperature. The expansion originates from strain pulses driven by an expansive laser-induced stress.^38^ Their propagation from the interfaces through the thin film with a sound velocity of $6.3\;\;\text{nm}\;\;{\text{ps}}^{-1}$^65^ causes the maximum expansion after 3 ps. The subsequent propagation of the strain pulses into the substrate reduces the expansion of the thin film. After 6 ps, the strain pulses have completely left the thin film due to the good acoustic impedance match between the SRO transducer and the STO substrate,^38^ and the remaining expansion is proportional to the laser-induced stress. The temperature-independent shape of the picosecond strain response indicates a sub-picosecond stress rise time for all stress components, i.e., from the electrons, phonons, and magnetic excitations. This is consistent with the temperature-independent phase of the oscillations in laser-excited SRO/STO superlattices.^29,66^ For temperatures below $T_{C}$, the reduced expansion, however, indicates an additional contractive magnetic stress resulting from optically induced magnetic excitations.

We extract the total laser-induced stress of the first pump pulse from the strain level in the time range from 6 to 9 ps and of the second pump pulse from the difference between the strain level in the time ranges from 6 to 9 ps and 16 to 20 ps. We estimate the uncertainty of the extracted stress by the standard deviation of the transient strain for these delays. The uncertainty in the strain determination originates from the small diffraction intensity of the thin SRO film and the intense underlying substrate Bragg peak that has to be subtracted. The extracted stress is depicted in Figs. 3(b) and 3(e), and its uncertainty is displayed by error bars.

In Fig. 3(b), the extracted temperature-dependent stress induced by the first high-fluence pump pulse decreases continuously below $T_{C}$ (blue dots and blue line). The second pump pulse induces a temperature-independent stress (red dots and red line) indicating the absence of a magnetic stress, because for all temperatures, the same amount of energy was deposited to the sample. We conclude that the first pulse saturates the magnetic excitations by a full demagnetization. This changes qualitatively for the low-fluence regime. The strain response to the low-fluence excitation [Fig. 3(e)] is nearly the same for the first (blue) and second pulse (red). In both cases, the stress decreases in a step-like fashion at the transition temperature. However, for the second pulse, the step is at a slightly lower starting temperature because the first pulse increases the temperature before the second pulse arrives.

## IV. MODELING THE TIME-RESOLVED EXPERIMENTS

Our modeling is simplified by the temperature-independent shape of the transient strain indicating a sub-picosecond rise of an expansive laser-induced total stress as superposition of contributions from phonons and magnetic excitations. Recent experiments on SRO observe an electron-phonon-coupling time of 130 fs determining the rise of the laser-induced stress.^38,66^ The ultrafast demagnetization of SRO as a measure of the energy transfer to the magnetic degrees of freedom is reported to occur in a two-step fashion within the first picosecond.^52,67^ The magnetic stress was shown to rise on the same timescale.^29^ Based on these results, we assume a distribution of energy to the phonons and the magnetic excitations as in equilibrium on a 200 fs timescale. Note, this assumption may be an oversimplification of the situation within the first femtoseconds in regards of non-thermal states of both the phonons^5–7^ and spins^68,69^ reported for various materials. However, the presented analysis does not provide insight into these details on femtosecond timescale because it is focused on the 6–9 ps timescale when the strain pulses have left the film.

Under this assumption, we model the temperature-dependent total stress after 6 ps by

$$\begin{array}{c} \sigma_{3}(T,z)=\sigma_{3,\text{ph}}(T,z)+\sigma_{3,\text{mag}}(T,z)\\ =\Gamma_{3,\text{ph}}\rho_{\text{ph}}^{Q}(T,z)+\Gamma_{3,\text{mag}}\rho_{\text{mag}}^{Q}(T,z), \end{array}$$ (3)

where the depth-dependent, deposited energy density $\rho_{r}^{Q}(z)=\int_{T}^{T^{\prime }(z)}C_{r}(T^{\prime \prime })dT^{\prime \prime }$ follows Lambert-Beers law. We calibrate the total absorbed energy density $\rho_{\text{dep}}^{Q}=\rho_{\text{ph}}^{Q}+\rho_{\text{mag}}^{Q}$ by the expansion in the paramagnetic phase, where $\rho_{\text{mag}}^{Q}=0$. This model neglects any heat diffusion to the substrate that is expected to be small for the considered time. Here, we use the Grüneisen constants and the phononic heat capacity of bulk SRO but take into account the magnetic heat capacity of the thin film accounting for the substrate-induced decrease in the transition temperature and broadening of the transition (see Sec. II).

We model the strain response to the stress in the SRO film using a one-dimensional linear-chain model of masses and springs that is provided by the modular Matlab library udkm1Dsim.^70^ This library, furthermore, translates the strained unit cells to the SRO Bragg peak shift using dynamical x-ray diffraction theory that is then analyzed in the same way as the UXRD data (Sec. II A). The modeled transient strain (solid lines) matches the amplitude and the shape of the strain response in Figs. 3(a) and 3(d).

Figures 3(b) and 3(e) show an agreement of our stress model provided by Eq. (3) (solid blue lines) with the extracted stress from the transient strain 6 ps after the excitation (blue dots). Equation (3) relates the temperature dependence of the laser-induced stress to the excitation of the magnetic subsystem. Full demagnetization may be expected if the phonon temperature exceeds the magnetic phase transition temperature.^71^ Since phonon and spin temperatures may be ill-defined at very early times, we chose to sketch in Figs. 3(c) and 3(f) how the magnetic heat capacity depends on the energy density. In thermal equilibrium, the sharp drop of the magnetic heat capacity occurs at *T_C_*. The deposition of energy in the nonequilibrium by the first and the second excitation is sketched by blue and red arrows, respectively, to illustrate when we can expect to saturate the spin excitations. In the case of the high fluence, the saturation of the spin system is already achieved with the first pulse. Therefore, the second laser pulse excites exclusively phonons in the fully demagnetized SRO layer resulting in a temperature-independent stress. This simplifies the stress induced by the first pulse from Eq. (3) to

$$\sigma_{3}^{\text{high}}(T)=\Gamma_{\text{pho},3}\rho_{\text{dep}}^{Q}+(\Gamma_{\text{mag},3}-\Gamma_{\text{pho},3})\rho_{\text{mag}}^{\text{high}}(T).$$ (4)

Here, the temperature-dependent magnetic energy density is given by the total integral of the remaining magnetic heat capacity $\rho_{\text{mag}}^{\text{high}}(T)=\int_{T}^{170\;K}C_{\text{mag}}^{\text{film}}(T\prime )\;dT\prime$. The energy density transferred to the magnetic system reduces the total stress by a contractive magnetic contribution and a reduced expansive phonon contribution due to the smaller energy density stored in the phonons. Above 170 K, all energy is transferred to the phonons (except the negligible energy density $\rho_{e}^{Q}$ of the electrons) and leads to a temperature-independent expansion. The molecular field approximation relates $\rho_{\text{mag}}^{\text{high}}(T)$ to the squared magnetization *M*^2^. The inherited temperature dependence of the total stress was also reported for SRO/STO-superlattices^29^ excited by a high fluence of $4.5\;\text{mJ}\;{\text{cm}}^{-2}$.

The temperature dependence changes with decreasing fluence as illustrated in Fig. 3(e). Since the magnetic heat capacity exhibits a peak at $T_{C}$, the magnetic stress contribution becomes maximal, when the deposited energy density corresponds to reaching the transition temperature. Increasing the initial temperature further saturates the magnetic stress and, thus, reduces the contractive stress contribution. This temperature-dependent saturation of the magnetic stress slightly below $T_{C}$ leads to the observed step-like behavior, where the step-width corresponds to the laser-induced increase in the phonon temperature at 6 to 9 ps. For the second pulse, the step-like behavior is shifted by 30 K corresponding to the temperature increase induced by the first pump pulse. In total, the temperature-dependent expansion follows the magnetic heat capacity and is given by Eq. (5) in the limit of infinitesimally small excitations,

$$\sigma_{3}^{\text{low}}(T)=\rho_{\text{dep}}V\left(\frac{\Gamma_{3,\text{mag}}C_{\text{mag}}(T)+\Gamma_{3,\text{ph}}C_{\text{ph}}(T)}{C_{\text{tot}}(T)}\right).$$ (5)

Here, the energy density of the subsystems is given by $\rho_{r}^{Q}=C_{r}\Delta T$ for a sufficiently small $\Delta T=\rho_{\text{dep}}/C_{\text{tot}}$.

**FIG. 4.**
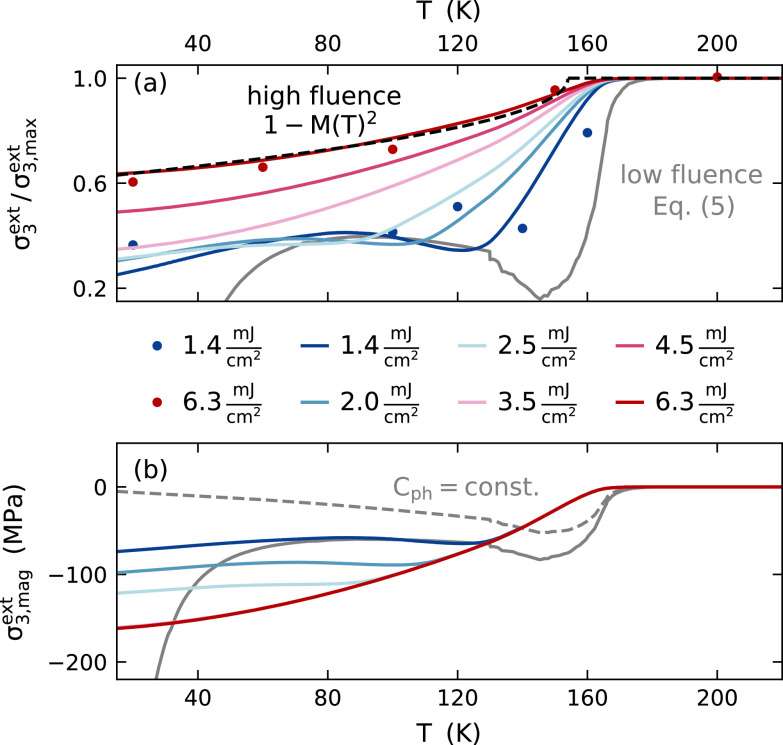
The fluence dependence of the temperature-dependent stress predicted by the Grüneisen model (3): the total stress (a) displays a continuous transition from the low fluence limit [Eq. (5)] to the high fluence limit [Eq. (4)], which is proportional to $1-M^{2}(T)$ (black dashed line). The extracted magnetic strain contribution (b) relates the minimum in total stress to a maximum of the contractive magnetic stress contribution corresponding to an enhanced fraction of deposited energy density. In (b), the dashed gray line depicts the magnetic contribution in case of a constant phononic heat capacity in the Doulong–Petit limit.

Figure 4(a) illustrates the crossover of the temperature dependence of the normalized stress according to Eq. (3) from the low fluence [Eq. (5)] to the high fluence limit [Eq. (4)]. With increasing fluence, the step-like change of the expansion, given by the magnetic heat capacity, becomes more continuous following a curve that is proportional to $1-M^{2}(T)$ for fluences above $3.5\;\;\text{mJ}\;\;{\text{cm}}^{-2}$. With increasing excitation fluence, the saturation of the magnetic stress occurs at lower initial temperatures as indicated by the shift of the maximum of the magnetic stress contribution $\sigma_{3,\text{mag}}^{\text{ext}}$ in Fig. 4(b). This maximum coincides with the maximum fraction of energy density stored in the magnetic system $\rho_{\text{mag}}^{Q}/\rho_{\text{dep}}^{Q}$ due to the peak in the magnetic heat capacity at $T_{C}$. The magnetic fraction of the heat capacity increases at very low temperatures, due to the different temperature dependence of the magnetic and phononic heat capacity. Therefore, the total stress in the limit of infinitesimally small excitation [Eq. (5)] in Fig. 4(a) differs from the shape of the magnetic heat capacity and vanishes at 40 K, where the products of heat capacities and Grüneisen constants for both subsystems are equal. If we assume a constant phonon heat capacity, the magnetic stress in the limit of infinitesimal excitations would, indeed, be given by the shape of the magnetic heat capacity as exemplified by the gray-dashed line in Fig. 4(b). This corresponds to the Dulong-Petit limit, which is realized in magnetic materials with $T_{C}>T_{D}$.

A detailed study that measures both magnetization dynamics and strain on the same sample under identical low and high fluence excitation may yield microscopic insight into the complex coupling of spin and lattice. However, to access the ultrafast timescale, ultrathin SRO layers have to be investigated, which calls for the high brilliance of a free-electron laser.

## V. CONCLUSION

In conclusion, we applied a thermodynamic model based on the concept of anisotropic Grüneisen constants to describe the fluence and temperature dependence of the laser-induced stresses in a thin SRO film. The total stress depends on the population of magnetic excitations, which is intrinsically limited by the temperature-dependent, finite integral of the magnetic heat capacity. The fluence dependence of the stress is explained by the saturation of the magnetic stress, which is observed by the picosecond strain response to a second pump pulse.

For the two limiting cases of small, laser-induced temperature increases and a complete transient demagnetization, we determine simplified descriptions of the total anisotropic lattice stress. For low fluences, the temperature-dependent expansion is given by the magnetic heat capacity $C_{\text{mag}}\propto M\partial M/\partial T$. For fluences saturating the magnetic stress, the temperature dependence is determined by the integrated magnetic heat capacity $\rho_{\text{mag}}^{\text{high}}\propto M{\left(T\right)}^{2}$ that we extract from the molecular field approximation.

We think that this analysis is helpful to estimate the strain induced by repeated heat load in SRO layers or similar materials with magnetic phase transitions. For SRO, the analysis is facilitated by the sub-picosecond rise of the stress contributions of phonons and magnetic excitations. Nonetheless, the expansion driven by an excitation faster than the nanosecond timescale of in-plane strain relaxation differs qualitatively from the equilibrium thermal expansion of bulk or thin film samples. This is due to the anisotropic nature of the magnetic stress and the vanishing out-of-plane Poisson contributions on the picosecond timescale. While, for our ${\left[001\right]}_{o}$-oriented film, the ultrafast suppression of the Poisson effect only changes the magnitude of the expansion [Fig. 2(c)], the bulk equilibrium expansion of a ${\left[001\right]}_{o}$-oriented SRO film predicts negative thermal expansion [Fig. 2(b)] although, according to our analysis, a time-resolved experiment would show a transient expansion because the Poisson effect prevails over the contractive magnetic stress.

## Data Availability

The data that support the findings of this study are available from the corresponding author upon reasonable request.

## References

[1] C. Thomsen , H. T. Grahn , H. J. Maris , and J. Tauc , “ Surface generation and detection of phonons by picosecond light pulses,” Phys. Rev. B 34, 4129 (1986). 10.1103/PhysRevB.34.4129 9940178

[2] O. Matsuda , M. C. Larciprete , R. L. Voti , and O. B. Wright , “ Fundamentals of picosecond laser ultrasonics,” Ultrasonics 56, 3 (2015). 10.1016/j.ultras.2014.06.005 24998119

[3] T. Barron , “ Grüneisen parameters for the equation of state of solids,” Ann. Phys. 1, 77 (1957). 10.1016/0003-4916(57)90006-4

[4] T. Barron , J. Collins , and G. White , “ Thermal expansion of solids at low temperatures,” Adv. Phys. 29, 609 (1980). 10.1080/00018738000101426

[5] L. Waldecker , R. Bertoni , R. Ernstorfer , and J. Vorberger , “ Electron-phonon coupling and energy flow in a simple metal beyond the two-temperature approximation,” Phys. Rev. X 6, 021003 (2016). 10.1103/PhysRevX.6.021003

[6] P. Maldonado , K. Carva , M. Flammer , and P. M. Oppeneer , “ Theory of out-of-equilibrium ultrafast relaxation dynamics in metals,” Phys. Rev. B 96, 174439 (2017). 10.1103/PhysRevB.96.174439

[7] P. Maldonado , T. Chase , A. Reid , X. Shen , R. Li , K. Carva , T. Payer , M. H. von Hoegen , K. Sokolowski-Tinten , X. Wang *et al.*, “ Tracking the ultrafast nonequilibrium energy flow between electronic and lattice degrees of freedom in crystalline nickel,” Phys. Rev. B 101, 100302 (2020). 10.1103/PhysRevB.101.100302

[8] A. Reid , X. Shen , P. Maldonado , T. Chase , E. Jal , P. Granitzka , K. Carva , R. Li , J. Li , L. Wu *et al.*, “ Beyond a phenomenological description of magnetostriction,” Nat. Commun. 9(1), 1035 (2018). 10.1038/s41467-018-03389-4 29515124 PMC5841346

[9] J. Vetelino , S. Mitra , and K. Namjoshi , “ Lattice dynamics, mode grüneisen parameters, and coefficient of thermal expansion of CsCl, CsBr, and CsI,” Phys. Rev. B 2, 2167 (1970). 10.1103/PhysRevB.2.2167

[10] J. Fabian and P. B. Allen , “ Thermal expansion and Grüneisen parameters of amorphous silicon: A realistic model calculation,” Phys. Rev. Lett. 79, 1885 (1997). 10.1103/PhysRevLett.79.1885

[11] S. Nie , X. Wang , H. Park , R. Clinite , and J. Cao , “ Measurement of the electronic Grüneisen constant using femtosecond electron diffraction,” Phys. Rev. Lett. 96, 025901 (2006). 10.1103/PhysRevLett.96.025901 16486599

[12] X. Wang , S. Nie , J. Li , R. Clinite , M. Wartenbe , M. Martin , W. Liang , and J. Cao , “ Electronic Grüneisen parameter and thermal expansion in ferromagnetic transition metal,” Appl. Phys. Lett. 92, 121918 (2008). 10.1063/1.2902170

[13] M. Nicoul , U. Shymanovich , A. Tarasevitch , D. von der Linde , and K. Sokolowski-Tinten , “ Picosecond acoustic response of a laser-heated gold-film studied with time-resolved x-ray diffraction,” Appl. Phys. Lett. 98, 191902 (2011). 10.1063/1.3584864

[14] A. von Reppert , R. Sarhan , F. Stete , J. Pudell , N. Del Fatti , A. Crut , J. Koetz , F. Liebig , C. Prietzel , and M. Bargheer , “ Watching the vibration and cooling of ultrathin gold nanotriangles by ultrafast x-ray diffraction,” J. Phys. Chem. C 120, 28894 (2016). 10.1021/acs.jpcc.6b11651

[15] J. Pudell , A. Maznev , M. Herzog , M. Kronseder , C. Back , G. Malinowski , A. von Reppert , and M. Bargheer , “ Layer specific observation of slow thermal equilibration in ultrathin metallic nanostructures by femtosecond x-ray diffraction,” Nat. Commun. 9(1), 3335 (2018). 10.1038/s41467-018-05693-5 30127415 PMC6102217

[16] J.-E. Pudell , M. Mattern , M. Hehn , G. Malinowski , M. Herzog , and M. Bargheer , “ Heat transport without heating?—An ultrafast x-ray perspective into a metal heterostructure,” Adv. Funct. Mater. 30, 2004555 (2020). 10.1002/adfm.202004555

[17] E. Grüneisen , “ Theorie des festen Zustandes einatomiger Elemente,” Ann. Phys. 344, 257 (1912). 10.1002/andp.19123441202

[18] G. White , “ Thermal expansion of magnetic metals at low temperatures,” Proc. Phys. Soc. 86, 159 (1965). 10.1088/0370-1328/86/1/320

[19] A. Lord, Jr. , “ Thermal expansion due to spin waves at low temperatures,” J. Phys. Chem. Solids 28, 517 (1967). 10.1016/0022-3697(67)90322-8

[20] E. Fawcett , “ Magnetic Grüneisen parameters in 3d transition metals,” Physica B 159, 12 (1989). 10.1016/S0921-4526(89)80046-8

[21] G. Barrera , J. Bruno , T. Barron , and N. Allan , “ Negative thermal expansion,” J. Phys.: Condens. Matter 17, R217 (2005). 10.1088/0953-8984/17/4/R03

[22] R. Grosse , P. Krause , M. Meissner , and A. Tausend , “ The coefficients of thermal expansion and the gruneisen functions of trigonal and amorphous selenium in the temperature range between 10K and 300K,” J. Phys. C: Solid State Phys. 11, 45 (1977). 10.1088/0022-3719/11/1/016

[23] G. White , “ Phase transitions and the thermal expansion of holmium,” J. Phys.: Condens. Matter 1, 6987 (1989). 10.1088/0953-8984/1/39/009

[24] L. McKeehan and P. Cioffi , “ Magnetostriction in permalloy,” Phys. Rev. 28, 146 (1926). 10.1103/PhysRev.28.146

[25] E. R. Callen and H. B. Callen , “ Static magnetoelastic coupling in cubic crystals,” Phys. Rev. 129, 578 (1963). 10.1103/PhysRev.129.578

[26] E. Callen and H. B. Callen , “ Magnetostriction, forced magnetostriction, and anomalous thermal expansion in ferromagnets,” Phys. Rev. 139, A455 (1965). 10.1103/PhysRev.139.A455

[27] M. Doerr , M. Rotter , and A. Lindbaum , “ Magnetostriction in rare-earth based antiferromagnets,” Adv. Phys. 54(1), 1 (2005). 10.1080/00018730500037264

[28] F. Darnell , “ Temperature dependence of lattice parameters for Gd, Dy, and Ho,” Phys. Rev. 130, 1825 (1963). 10.1103/PhysRev.130.1825

[29] C. von Korff-Schmising , A. Harpoeth , N. Zhavoronkov , Z. Ansari , C. Aku-Leh , M. Woerner , T. Elsaesser , M. Bargheer , M. Schmidbauer , I. Vrejoiu *et al.*, “ Ultrafast magnetostriction and phonon-mediated stress in a photoexcited ferromagnet,” Phys. Rev. B 78, 060404 (2008). 10.1103/PhysRevB.78.060404

[30] S. O. Mariager , F. Pressacco , G. Ingold , A. Caviezel , E. Möhr-Vorobeva , P. Beaud , S. Johnson , C. Milne , E. Mancini , S. Moyerman *et al.*, “ Structural and magnetic dynamics of a laser induced phase transition in FeRh,” Phys. Rev. Lett. 108, 087201 (2012). 10.1103/PhysRevLett.108.087201 22463562

[31] A. von Reppert , L. Willig , J.-E. Pudell , S. Zeuschner , G. Sellge , F. Ganss , O. Hellwig , J. Arregi , V. Uhlíř , A. Crut *et al.*, “ Spin stress contribution to the lattice dynamics of FePt,” Sci. Adv. 6, eaba1142 (2020). 10.1126/sciadv.aba1142 32685678 PMC7343378

[32] T. Kiyama , K. Yoshimura , K. Kosuge , Y. Ikeda , and Y. Bando , “ Invar effect of SrRuO_3_: Itinerant electron magnetism of Ru 4d electrons,” Phys. Rev. B 54, R756 (1996). 10.1103/PhysRevB.54.R756 9985427

[33] S. Bushmeleva , V. Y. Pomjakushin , E. Pomjakushina , D. Sheptyakov , and A. Balagurov , “ Evidence for the band ferromagnetism in SrRuO_3_ from neutron diffraction,” J. Magn. Magn. Mater. 305, 491 (2006). 10.1016/j.jmmm.2006.02.089

[34] B. Dabrowski , M. Avdeev , O. Chmaissem , S. Kolesnik , P. Klamut , M. Maxwell , and J. Jorgensen , “ Freezing of octahedral tilts below the curie temperature in SrRu_1−*v*_O_3_ perovskites,” Phys. Rev. B 71, 104411 (2005). 10.1103/PhysRevB.71.104411

[35] A. von Reppert , J. Pudell , A. Koc , M. Reinhardt , W. Leitenberger , K. Dumesnil , F. Zamponi , and M. Bargheer , “ Persistent nonequilibrium dynamics of the thermal energies in the spin and phonon systems of an antiferromagnet,” Struct. Dyn. 3, 054302 (2016). 10.1063/1.4961253 27679803 PMC5018005

[36] J. Pudell , A. von Reppert , D. Schick , F. Zamponi , M. Rössle , M. Herzog , H. Zabel , and M. Bargheer , “ Ultrafast negative thermal expansion driven by spin disorder,” Phys. Rev. B 99, 094304 (2019). 10.1103/PhysRevB.99.094304

[37] A. von Reppert , M. Mattern , J.-E. Pudell , S. Zeuschner , K. Dumesnil , and M. Bargheer , “ Unconventional picosecond strain pulses resulting from the saturation of magnetic stress within a photoexcited rare earth layer,” Struct. Dyn. 7, 024303 (2020). 10.1063/1.5145315 32232076 PMC7101248

[38] D. Schick , M. Herzog , A. Bojahr , W. Leitenberger , A. Hertwig , R. Shayduk , and M. Bargheer , “ Ultrafast lattice response of photoexcited thin films studied by x-ray diffraction,” Struct. Dyn. 1, 064501 (2014). 10.1063/1.4901228 26798784 PMC4714650

[39] A. Bojahr , M. Gohlke , W. Leitenberger , J. Pudell , M. Reinhardt , A. von Reppert , M. Roessle , M. Sander , P. Gaal , and M. Bargheer , “ Second harmonic generation of nanoscale phonon wave packets,” Phys. Rev. Lett. 115, 195502 (2015). 10.1103/PhysRevLett.115.195502 26588396

[40] M. Sander , M. Herzog , J.-E. Pudell , M. Bargheer , N. Weinkauf , M. Pedersen , G. Newby , J. Sellmann , J. Schwarzkopf , V. Besse *et al.*, “ Spatiotemporal coherent control of thermal excitations in solids,” Phys. Rev. Lett. 119, 075901 (2017). 10.1103/PhysRevLett.119.075901 28949697

[41] K. Wang , B. Zhang , W. Xie , S. Liu , X. Wei , Z. Cai , M. Gu , Y. Tao , T. Yang , C. Zhang *et al.*, “ Coupling among carriers and phonons in femtosecond laser pulses excited SrRuO_3_: A promising candidate for optomechanical and optoelectronic applications,” ACS Appl. Nano Mater. 2, 3882 (2019). 10.1021/acsanm.9b00728

[42] W. Tian , J. Haeni , D. G. Schlom , E. Hutchinson , B. Sheu , M. Rosario , P. Schiffer , Y. Liu , M. A. Zurbuchen , and X. Pan , “ Epitaxial growth and magnetic properties of the first five members of the layered ${\text{Sr}}_{n+1}$ Ru_n_O_3*n*+1_ oxide series,” Appl. Phys. Lett. 90, 022507 (2007). 10.1063/1.2430941

[43] G. Laskin , H. Wang , H. Boschker , W. Braun , V. Srot , P. A. van Aken , and J. Mannhart , “ Magnetic properties of epitaxially grown SrRuO_3_ nanodots,” Nano Lett. 19, 1131 (2019). 10.1021/acs.nanolett.8b04459 30645131 PMC6728099

[44] Q. Gan , R. Rao , C. Eom , J. Garrett , and M. Lee , “ Direct measurement of strain effects on magnetic and electrical properties of epitaxial SrRuO_3_ thin films,” Appl. Phys. Lett. 72, 978 (1998). 10.1063/1.120603

[45] I. Vrejoiu , M. Alexe , D. Hesse , and U. Gösele , “ Functional perovskites—From epitaxial films to nanostructured arrays,” Adv. Funct. Mater. 18, 3892 (2008). 10.1002/adfm.200800560

[46] C.-B. Eom , R. J. Cava , R. Fleming , J. M. Phillips , J. Marshall , J. Hsu , J. Krajewski , W. Peck *et al.*, “ Single-crystal epitaxial thin films of the isotropic metallic oxides ${\text{Sr}}_{1-x}$ Ca_x_RuO_3_ (0<x<1),” Science 258, 1766 (1992). 10.1126/science.258.5089.1766 17831659

[47] J.-P. Maria , H. McKinstry , and S. Trolier-McKinstry , “ Origin of preferential orthorhombic twinning in SrRuO_3_ epitaxial thin films,” Appl. Phys. Lett. 76, 3382 (2000). 10.1063/1.126654

[48] D. Schick , R. Shayduk , A. Bojahr , M. Herzog , C. von Korff-Schmising , P. Gaal , and M. Bargheer , “ Ultrafast reciprocal-space mapping with a convergent beam,” J. Appl. Crystallography 46, 1372 (2013). 10.1107/S0021889813020013

[49] L. Maerten , A. Bojahr , M. Gohlke , M. Rössle , and M. Bargheer , “ Coupling of ghz phonons to ferroelastic domain walls in SrTiO_3_,” Phys. Rev. Lett. 114, 047401 (2015). 10.1103/PhysRevLett.114.047401 25679906

[50] I. Mazin and D. J. Singh , “ Electronic structure and magnetism in Ru-based perovskites,” Phys. Rev. B 56, 2556 (1997). 10.1103/PhysRevB.56.2556

[51] G. Cao , S. McCall , M. Shepard , J. Crow , and R. Guertin , “ Thermal, magnetic, and transport properties of single-crystal ${\text{Sr}}_{1-x}$ Ca_x_RuO_3_ (0<x<1),” Phys. Rev. B 56, 321 (1997). 10.1103/PhysRevB.56.321

[52] T. Ogasawara , K. Ohgushi , Y. Tomioka , K. Takahashi , H. Okamoto , M. Kawasaki , and Y. Tokura , “ General features of photoinduced spin dynamics in ferromagnetic and ferrimagnetic compounds,” Phys. Rev. Lett. 94, 087202 (2005). 10.1103/PhysRevLett.94.087202 15783924

[53] M. Langner , C. Kantner , Y. Chu , L. Martin , P. Yu , J. Seidel , R. Ramesh , and J. Orenstein , “ Observation of ferromagnetic resonance in SrRuO_3_ by the time-resolved magneto-optical kerr effect,” Phys. Rev. Lett. 102, 177601 (2009). 10.1103/PhysRevLett.102.177601 19518833

[54] L. Klein , J. Dodge , C. Ahn , J. Reiner , L. Mieville , T. Geballe , M. Beasley , and A. Kapitulnik , “ Transport and magnetization in the badly metallic itinerant ferromagnet,” J. Phys.: Condens. Matter 8, 10111 (1996). 10.1088/0953-8984/8/48/026 10062042

[55] Q. Gan , R. Rao , C. Eom , L. Wu , and F. Tsui , “ Lattice distortion and uniaxial magnetic anisotropy in single domain epitaxial (110) films of SrRuO_3_,” J. Appl. Phys. 85, 5297 (1999). 10.1063/1.369859

[56] P. Allen , H. Berger , O. Chauvet , L. Forro , T. Jarlborg , A. Junod , B. Revaz , and G. Santi , “ Transport properties, thermodynamic properties, and electronic structure of SrRuO_3_,” Phys. Rev. B 53, 4393 (1996). 10.1103/PhysRevB.53.4393 9983992

[57] D. J. Singh , “ Electronic and magnetic properties of the 4d itinerant ferromagnet SrRuO_3_,” J. Appl. Phys. 79, 4818 (1996). 10.1063/1.361618

[58] E. H. P. Cordfunke , R. Van Der Laan , and J. Van Miltenburg , “ Thermophysical and thermochemical properties of BaO and SrO from 5 to 1000K,” J. Phys. Chem. Solids 55, 77 (1994). 10.1016/0022-3697(94)90186-4

[59] E. Cordfunke , R. Konings , E. Westrum, Jr. , and R. Shaviv , “ The thermophysical and thermochemical properties of RuO_2_ from 0 to 1000K,” J. Phys. Chem. Solids 50, 429 (1989). 10.1016/0022-3697(89)90444-7

[60] G. M. Leitus , S. Reich , and F. Frolow , “ Structural rearrangement in SrRuO_3_ near the magnetic critical point,” J. Magn. Magn. Mater. 206, 27 (1999). 10.1016/S0304-8853(99)00503-X

[61] J. M. D. Coey , *Magnetism and Magnetic Materials*, reprinted ed. ( Cambridge University Press, Cambridge, 2013).

[62] F. Bern , M. Ziese , A. Setzer , E. Pippel , D. Hesse , and I. Vrejoiu , “ Structural, magnetic and electrical properties of SrRuO_3_ films and SrRuO_3_/SrTiO_3_ superlattices,” J. Phys.: Condens. Matter 25, 496003 (2013). 10.1088/0953-8984/25/49/496003 24184982

[63] P. Kostic , Y. Okada , N. Collins , Z. Schlesinger , J. Reiner , L. Klein , A. Kapitulnik , T. Geballe , and M. Beasley , “ Non-Fermi-liquid behavior of SrRuO_3_: Evidence from infrared conductivity,” Phys. Rev. Lett. 81, 2498 (1998). 10.1103/PhysRevLett.81.2498

[64] D. Schick , A. Bojahr , M. Herzog , C. v Korff-Schmising , R. Shayduk , W. Leitenberger , P. Gaal , and M. Bargheer , “ Normalization schemes for ultrafast x-ray diffraction using a table-top laser-driven plasma source,” Rev. Sci. Instrum. 83, 025104 (2012). 10.1063/1.3681254 22380122

[65] S. Yamanaka , T. Maekawa , H. Muta , T. Matsuda , S-i Kobayashi , and K. Kurosaki , “ Thermophysical properties of SrHfO_3_ and SrRuO_3_,” J. Solid State Chem. 177, 3484 (2004). 10.1016/j.jssc.2004.05.039

[66] A. Bojahr , D. Schick , L. Maerten , M. Herzog , I. Vrejoiu , C. von Korff-Schmising , C. Milne , S. L. Johnson , and M. Bargheer , “ Comparing the oscillation phase in optical pump-probe spectra to ultrafast x-ray diffraction in the metal-dielectric SrRuO_3_/SrTiO_3_ superlattice,” Phys. Rev. B 85, 224302 (2012). 10.1103/PhysRevB.85.224302

[67] C. Kantner , M. Langner , W. Siemons , J. Blok , G. Koster , A. J. Rijnders , R. Ramesh , and J. Orenstein , “ Determination of the spin-flip time in ferromagnetic SrRuO_3_ from time-resolved kerr measurements,” Phys. Rev. B 83, 134432 (2011). 10.1103/PhysRevB.83.134432

[68] P. Tengdin , W. You , C. Chen , X. Shi , D. Zusin , Y. Zhang , C. Gentry , A. Blonsky , M. Keller , P. M. Oppeneer *et al.*, “ Critical behavior within 20 fs drives the out-of-equilibrium laser-induced magnetic phase transition in nickel,” Sci. Adv. 4, eaap9744 (2018). 10.1126/sciadv.aap9744 29511738 PMC5834307

[69] D. Zahn , F. Jakobs , Y. W. Windsor , H. Seiler , T. Vasileiadis , T. A. Butcher , Y. Qi , D. Engel , U. Atxitia , J. Vorberger *et al.*, “ Lattice dynamics and ultrafast energy flow between electrons, spins, and phonons in a 3d ferromagnet,” arXiv:2008.04611 (2020).

[70] D. Schick , A. Bojahr , M. Herzog , R. Shayduk , C. von Korff-Schmising , and M. Bargheer , “ udkm1Dsim—A simulation toolkit for 1D ultrafast dynamics in condensed matter,” Comput. Phys. Commun. 185, 651 (2014). 10.1016/j.cpc.2013.10.009

[71] W. You , P. Tengdin , C. Chen , X. Shi , D. Zusin , Y. Zhang , C. Gentry , A. Blonsky , M. Keller , P. M. Oppeneer *et al.*, “ Revealing the nature of the ultrafast magnetic phase transition in ni by correlating extreme ultraviolet magneto-optic and photoemission spectroscopies,” Phys. Rev. Lett. 121, 077204 (2018). 10.1103/PhysRevLett.121.077204 30169091

